# Exosomal CircHIPK3 Released from Hypoxia-Induced Cardiomyocytes Regulates Cardiac Angiogenesis after Myocardial Infarction

**DOI:** 10.1155/2020/8418407

**Published:** 2020-07-13

**Authors:** Yan Wang, Ranzun Zhao, Changyin Shen, Weiwei Liu, Jinson Yuan, Chaofu Li, Wenwen Deng, Zhenglong Wang, Wei Zhang, Junbo Ge, Bei Shi

**Affiliations:** ^1^Department of Cardiology, Affiliated Hospital of Zunyi Medical University, Zunyi 563000, China; ^2^Department of Cardiology, The Second Affiliated Hospital of Zunyi Medical University, Zunyi 563000, China; ^3^Department of Cardiology, Shanghai Institute of Cardiovascular Diseases, Zhongshan Hospital, Fudan University, Shanghai 200032, China

## Abstract

Exosomes play critical roles in mediating cell-to-cell communication by delivering noncoding RNAs (including miRNAs, lncRNAs, and circRNAs). Our previous study found that cardiomyocytes (CMs) subjected to hypoxia released circHIPK3-rich exosomes to regulate oxidative stress damage in cardiac endothelial cells. However, the role of exosomes in regulating angiogenesis after myocardial infarction (MI) remains unknown. The aim of this study was to establish the effects of exosomes derived from hypoxia-induced CMs on the migration and angiogenic tube formation of cardiac endothelial cells. Here, we reported that hypoxic exosomes (HPC-exos) can effectively reduce the infarct area and promote angiogenesis in the border surrounding the infarcted area. HPC-exos can also promote cardiac endothelial cell migration, proliferation, and tube formation in vitro. However, these effects were weakened after silencing circHIPK3 in hypoxia-induced CMs. We further verified that silencing and overexpressing circHIPK3 changed cardiac endothelial cell proliferation, migration, and tube formation in vitro by regulating the miR-29a expression. In addition, exosomal circHIPK3 derived from hypoxia-induced CMs first led to increased VEGFA expression by inhibiting miR-29a activity and then promoted accelerated cell cycle progression and proliferation in cardiac endothelial cells. Overexpression of miR-29a mimicked the effect of silencing circHIPK3 on cardiac endothelial cell activity in vitro. Thus, our study provides a novel mechanism by which exosomal circRNAs are involved in the communication between CMs and cardiac endothelial cells.

## 1. Introduction

It is important to regulate and maintain cardiac function by ensuring sufficient blood supply to deprived areas after myocardial infarction (MI) [1]. The maintenance of anatomic and functional integrity of the microvasculature after MI is dependent on the proliferation and migration of cardiac endothelial cells and neovascularization. It is well recognized that there is a direct path of communication between cardiomyocytes (CMs) and cardiac endothelial cells in the mammalian heart [2]. We and others have observed that exosomes derived from CMs contain a variety of miRNAs, circRNAs and proteins, which may be transferred to adjacent endothelial cells and consequently regulate their function [3, 4].

Exosomes are involved in regulating the function of target cells by releasing their contents into the target cells [5]. Therefore, exosomes can induce completely different outcomes in recipient cells since the composition of exosomes changes depending on the physiological state of the producing cell [6, 7]. CMs, as well as many other types of cells, can release exosomes [8, 9]. Recently, CM-derived exosomes were found to promote angiogenesis by delivering miR-222 under ischemic conditions [10]. In this context, we demonstrated that exosomes derived from CMs cultured under hypoxic conditions are able to protect endothelial cells from H_2_O_2_-induced apoptosis and that this effect was dependent on the delivery of circHIPK3 [11]. However, the effect of exosomal circHIPK3 released by hypoxia-induced CMs on the proliferation and migration cardiac endothelial cells and neovascularization remains to be elucidated.

As one of the most abundant circRNAs in the heart [12], circHIPK3 has previously been confirmed to be involved in mediating a wide range of physiological and pathological processes, such as cell survival, autophagy, proliferation, and angiogenesis, by sponging different miRNAs [13–16]. In the present study, we demonstrated in vitro and in vivo that the exosomal circHIPK3 released by hypoxia-induced CMs stimulates cardiac angiogenesis in MI via miR-29a-mediated regulation of VEGFA.

## 2. Materials and Methods

### 2.1. Animals

This study conforms to the Guide for the Care and Use of Laboratory Animals in China. All experimental procedures were in accordance with the protocols approved by the Institutional Animal Care and Use Committee of Zunyi Medical University. Three-week-old wild-type (WT) C57BL/6J mice were procured from Zunyi Medical University (Zunyi, China).

### 2.2. Hypoxic Preconditioning of CMs

Mouse CMs were cultured as we previously described [11]. Briefly, neonatal mice were sacrificed after heparinization and were sterilized. Trypsin (0.03%, Sigma) and collagenase type II (0.04%, Sigma) were used to digest the ventricle fragments. Subsequently, mouse CMs were purified by differential attachment culture to remove cardiac fibroblasts. Afterwards, CMs were subjected to hypoxia. Approximately 5 × 10^6^ CMs were incubated in complete Dulbecco's modified Eagle medium supplemented with 10% fetal calf serum (FBS) under a 94% N_2_, 5% CO_2_, and 1% O_2_ gas mixture in an incubator (Galaxy Corporation, USA) at 37°C for 12 h.

### 2.3. Cardiac Endothelial Cell Culture and Establishment of the H_2_O_2_ Oxidative Stress Model

The isolation of cardiac endothelial cells was performed according to a previously published protocol [17]. Briefly, mice were euthanized by an overdose of Avertin (200 mg/kg). LV tissue was dissected into ≈1 mm^3^ pieces and subsequently digested in 450 U/mL collagenase I, 60 U/mL DNase I, and 60 U/mL hyaluronidase (Sigma-Aldrich) for 1 h at 37°C under agitation (750 rpm). The cells were then filtered through a 40 *μ*m nylon mesh (BD Falcon), washed, and centrifuged (8 min, 300 × g, 4°C). A magnetic bead separation assay with CD31^+^ beads was used to isolate cardiac endothelial cells, and the purity of the cell population was confirmed by FACS analysis of CD31^+^ (clone 390) cells. To establish an oxidative stress model for subsequent experiments, these cells were exposed to 200 *μ*M H_2_O_2_ for 3 h as previously described [18].

### 2.4. Isolation and Internalization of Exosomes

The extraction of CM-derived exosomes was performed as previously described [19]. After CMs were subjected to normoxic or hypoxic conditions for 12 h, the media were collected to obtain exosomes by ultracentrifugation (Beckman Coulter SW 41 Ti rotor and an XPN-100 ultracentrifuge). Then, the exosomes were added to cardiac endothelial cells for update [7] by incubating 300 *μ*g/mL exosomes with cardiac endothelial cells for 24 h [11].

### 2.5. CircRNA-miRNA Interaction

Interactions between circHIPK3 and miR-29a were predicted using proprietary miRNA target prediction software from Arraystar based on miRanda and TargetScan. Our previous study confirmed that circHIPK3 and miR-29a colocalize in the cytoplasm and have binding sites to each other [11].

### 2.6. Cell Transfection

CircHIPK3, small interfering RNAs (siRNAs) against circHIPK3, the linear HIPK3 gene, and siRNAs against HIKP3 were transfected into CMs or cardiac endothelial cells using a lentiviral construct according to the manufacturer's protocol (Hanbio, China) and performed as previously published [11]. In addition, miR-29a mimic, inhibitor, or their corresponding negative controls (RiboBio, China) were transfected into cardiac endothelial cells with Lipofectamine 3000 (Invitrogen, USA) according to the kit's instructions. At 48 h after transfection, qPCR was used to evaluate the gene expression in cardiac endothelial cells.

### 2.7. Real-Time qPCR

The relative expression of circHIPK3, miR-29a, and VEGFA mRNA was measured as previously described [11]. TRIzol reagent (Life Technologies, Carlsbad, CA) was used to isolate total RNA from cell lysates, after which 500 ng of RNA was subjected to reverse transcription with a PrimeScript RT Master Mix (Takara, Dalian, China) to synthesize cDNAs from circHIPK3, miR29a, and VEGFA mRNA. qPCR analyses of the cDNAs were performed with SYBR Premix Ex Taq II (Takara). RNase R treatment was used for circRNA detection as before. The amount of miRNA was detected using Stem-loop qPCR TaqMan MicroRNA assays (Life Technologies), and GAPDH and U6 were used as the internal reference for mRNA and miRNA expression, respectively. The 2^−*∆∆*Ct^ method was used to quantify gene expression. All primer sequences are listed in Table 1 and were designed and synthesized by RiboBio (Guangzhou, China).

### 2.8. Western Blot

The protein levels in cardiac endothelial cells were analyzed by Western blot as previously described. Protein extracts were separated by SDS-PAGE and transferred onto PVDF membranes. After the membranes were blocked overnight in BSA solution, they were probed with primary antibodies against VEGFA, CyclinD1, PCNA, and *β*-actin. Next, GAPDH and horseradish peroxidase-conjugated secondary antibodies were incubated with the PVDF membranes for 1 h, and then, an enhanced chemiluminescence reagent (Amersham Biosciences, USA) was incubated on the PVDF membranes before the protein band intensities were detected with a ChemiDoc MP system.

### 2.9. Cardiac Endothelial Cell Proliferation, Migration, and Tube Formation Assays

Cell migration was determined using Transwell assays as previously described [20]. A total of 100 *μ*L of cell suspension (1 × 10^5^ cells/mL) was added to the upper chamber, and 600 *μ*L of complete medium was added to the lower chamber. After 24 h, cells that did not migrate to the lower chamber were removed. Subsequently, the chamber was fixed in 4% paraformaldehyde for 30 min and dyed with 1% crystal violet for another 15 min. Five randomly selected fields in each chamber were imaged with an inverted microscope (magnification 10x) to determine the number of migrated cells.

Cell proliferation was detected with the Cell-Light 5-Ethynyl-20-deoxyuridine (EdU) Kit (RiboBio, China) according to the manufacturer's protocol. After cells were incubated with EdU for 2 h, they were stained with Apollo Dye Solution for 30 min followed by Hoechst 33342 staining. EdU-positive cells in five randomly selected fields were imaged and counted under an Olympus FSX100 microscope (Olympus, Tokyo, Japan).

Cardiac endothelial cells (1 × 10^5^ cells/mL) were plated on 120 *μ*L of Matrigel (BD Falcon) in a 48-well plate and incubated at 37°C in an atmosphere of 5% CO2 for 16 h. The gels were observed under an inverted microscope (4x magnification) (Nikon TS100), and the numbers of branch points and tubes formed for each unique tube structure were counted in each image.

### 2.10. Cell Cycle Assay

Cardiac endothelial cells were harvested and then washed with ice-cold phosphate-buffered saline (PBS). The cells were fixed in 70% ethanol for 24 h before they were resuspended in 500 *μ*L of PBS containing 10 *μ*g/mL RNase A, 50 *μ*g/mL propidium iodide (Sigma), 0.1% Triton X-100, and 0.1% sodium citrate. The suspensions were incubated for 15 min at room temperature in the dark and then immediately analyzed on a flow cytometer (Millipore Guava).

### 2.11. MI Induction and Exosome Delivery

Mice underwent inhalational anesthesia with 2% isoflurane and were subjected to MI via ligation of the left anterior descending coronary artery (LAD) as described previously [21, 22]. Immediately after LAD ligation, the mice received an intramyocardial injection of either 1 *μ*g/g exosomes or PBS (*n* = 10) in a total volume of 20 *μ*L at 5 different sites in the peri-infarct area. All mice were followed up at 4 weeks post-MI to evaluate both LV functional changes using echocardiography and structural remodeling.

### 2.12. Echocardiography

Echocardiography was performed on mice before MI (baseline) and at 4 weeks post-MI using a Vevo 770 imaging system (VisualSonics, Toronto, Canada) equipped with a 30 MHz transducer. The mice underwent inhalational anesthesia with a mixture of 1.5% isoflurane and oxygen (1 L/min). The internal diameter of the LV was measured in the short-axis view from M-mode recordings, and the ejection fraction (EF) and left ventricular end diastolic diameter (LVIDd) were calculated using corresponding formulas as previously described [23].

### 2.13. Morphometric Studies

Mice were euthanized by an overdose of Avertin (200 mg/kg). The hearts were fixed with 10% buffered formalin and paraffin embedded. Morphometric analysis was performed on tissue sections prepared with Masson's trichrome and hematoxylin/eosin staining using ImageJ software. The fibrotic area was measured to determine the percentage of fibrosis [17].

### 2.14. Histology

Immunofluorescence staining of tissue sections was performed as described previously [24]. The formation of a new capillary network was assessed by CD31 (Abcam) staining as described before [17]. Nuclei were counterstained with 4,6-diamidino-2-phenylindole (Sigma-Aldrich, St. Louis, MO), and 10 randomly selected fields in each sample were imaged under a fluorescence microscope (Olympus, Tokyo, Japan).

### 2.15. Statistical Analysis

Analyses were conducted with the SPSS 21.0 statistical package (IBM, Armonk, NY, USA). The data were normally distributed and are expressed as the mean ± SD. Comparisons among multiple groups were performed with one-way analysis of variance (ANOVA). In addition, a *P* value < 0.05 was considered statistically significant.

## 3. Results

### 3.1. HPC-Exos Promote Angiogenesis following MI In Vivo

**Figure 1:**
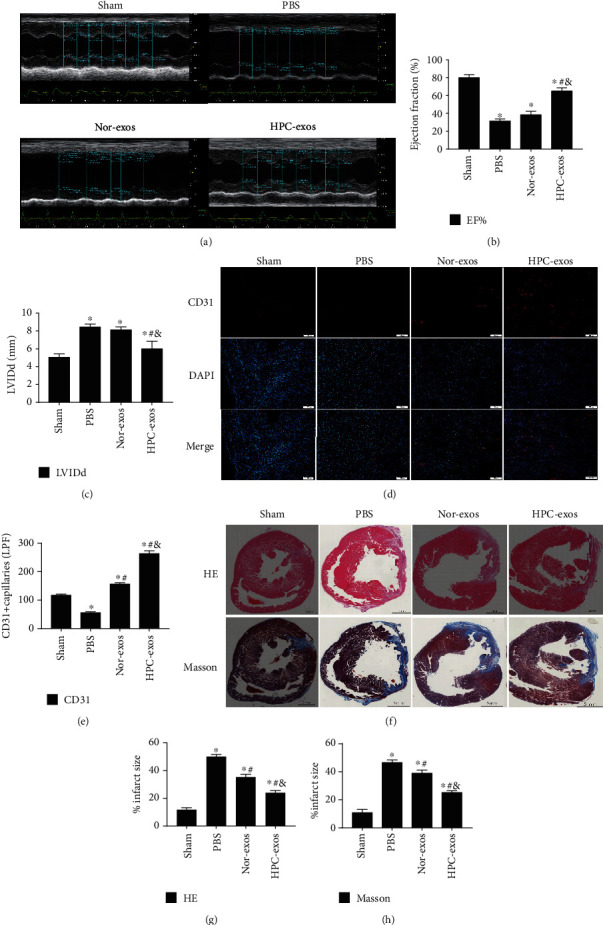
HPC-exos stimulate neovascularization and improve cardiac function at 4 weeks following MI. PBS, Nor-exos, and HPC-exos were delivered to the border area of MI at the moment of injury. Four weeks following MI, the neovascularization density, cardiac function, and fibrosis levels were evaluated. (a) Representative echocardiographic images. The LVEF% (b) and LVIDd (c) were calculated in various groups as indicated (*n* = 5 for the PBS group and *n* = 6 for the Nor-exo and HPC-exo groups). (d) CD31 immunolabeling in the infarcted area. (e) Quantification of CD31-positive cells. (f) Representative cross-sectional images of hearts subjected to hematoxylin-eosin staining and Masson's trichrome staining 4 weeks after exosome transplantation. The blue color indicates fibrosis, and the red color indicates myocardial fibers. (g, h) HPC-exo transplantation reduced the fibrotic area (mean ± SD, *n* = 3; ^∗^*P* < 0.05, compared with the sham group, ^#^*P* < 0.05 compared with the PBS group, and ^&^*P* < 0.05 compared with the Nor-exo group).

To evaluate the beneficial function of exosomes released from hypoxic CMs, normoxic exosomes (Nor-exos), or hypoxic exosomes (HPC-exos) were delivered to the border area of MI at the moment of injury. Four weeks after MI, mice administered with HPC-exos tended to have an increased EF and decreased LVIDd (Figures 1(a)–1(c)). Most importantly, the myocardial vascular density was increased in the infarcted region in mice in the HPC-exo group, as shown by the increase in CD31-stained cells (Figures 1(d) and 1(e)). CM-derived exosomes may also reduce cardiac fibrosis and improve ventricular remodeling after MI [10]. Masson's and hematoxylin-eosin staining showed a substantial decrease in myocardial fibrosis after HPC-exo treatment compared with PBS treatment in mice subjected to MI (Figures 1(f)–1(h)). Altogether, these results indicated that the exosomes produced by hypoxic CMs promote neovascularization after MI and ameliorate myocardial fibrosis.

### 3.2. HPC-Exos Promote Cardiac Endothelial Cell Migration, Proliferation, and Tube Formation

**Figure 2:**
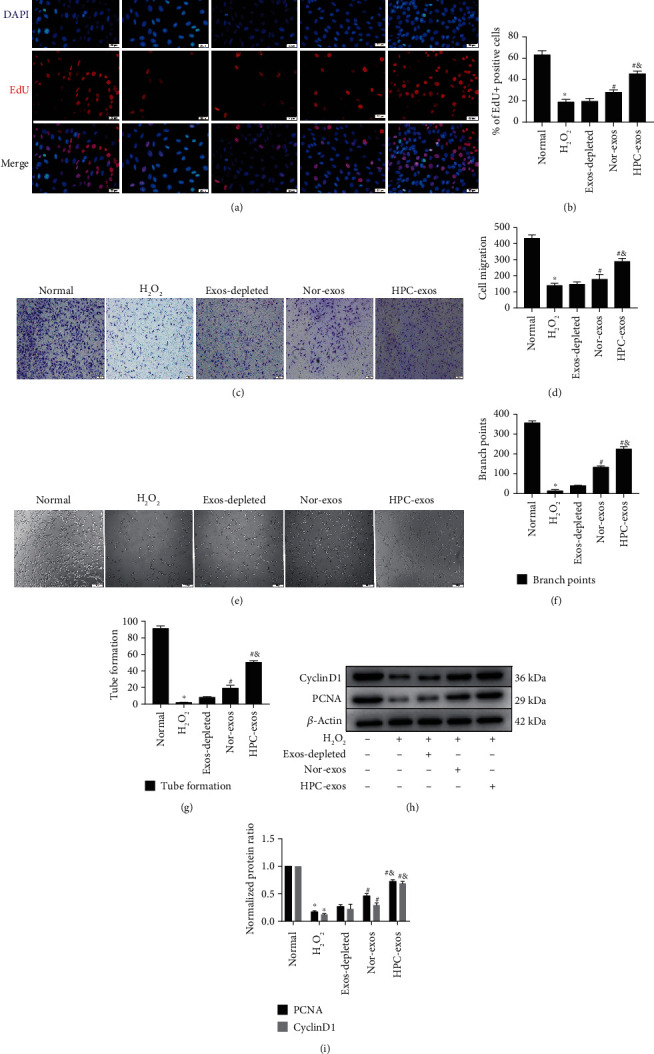
HPC-exos promote cardiac endothelial cell migration, proliferation, and tube formation. Cardiac endothelial cells were incubated with depleted exos, Nor-exos, or HPC-exos for 24 h after exposure to 200 *μ*M H_2_O_2_ for 3 h. (a) Cell proliferation was detected by using the EdU assay. (b) Quantification of the data presented in (a). (c) The migration ability of cardiac endothelial cells was measured by Transwell assay (original magnification, ×200). (d) Quantification of the data presented in (c). (e) Tube formation in Matrigel was assessed. (f, g) Quantitative assessment of the number of branch points and tubes formed. (h) The protein expression levels of cyclinD1 and PCNA were determined using Western blot analysis. (i) Quantification of the relative expression levels of cyclinD1 and PCNA. The results are normalized to the control (mean ± SD, *n* = 3; ^∗^*P* < 0.05, compared with the normal group; ^#^*P* < 0.05 compared with the H_2_O_2_ group; and ^&^*P* < 0.05 compared with the Nor-exo group).

As a multistep physiological process, angiogenesis comprises several sequential steps involving the proliferation, migration, and morphogenesis of cardiac endothelial cells. Therefore, we tested whether CM-derived exosomes impact the behavior and angiogenic potential capacity of cardiac endothelial cells in vitro. H_2_O_2_ (200 *μ*M for 3 h) was used to pretreat cardiac endothelial cells to simulate the microenvironment of oxidative stress in vitro. The results demonstrated that, compared with the normal group, the H_2_O_2_ group showed decreased proliferation, migration, and tube formation (Figures 2(a)–2(i)). Furthermore, compared with Nor-exos, HPC-exos promoted cardiac endothelial cell proliferation and migration after 24 h of treatment, as indicated by the EdU and Transwell assays (Figures 2(a)–2(d)). CyclinD1 and PCNA are proliferation markers and are required for regulating the cell cycle of proliferating endothelial cells at G1 phase [25]. Western blot assays also indicated that cyclinD1 and PCNA were upregulated in the HPC-exo group compared with the Nor-exo group (Figures 2(h)–2(i)). Moreover, compared to Nor-exos, HPC-exos exhibited an increased capacity to induce tube formation, as demonstrated by the presence of more branch points and tube-like segments in the Matrigel assay (Figures 2(e)–2(g)). Taken together, these results provide credible evidence that HPC-exos promote cardiac endothelial cell migration, proliferation, and tube formation, which are the cardinal features of angiogenesis.

### 3.3. HPC-Exosomal CircHIPK3 Is Involved in Promoting the Cell Cycle and Accelerating Migration in Cardiac Endothelial Cells

**Figure 3:**
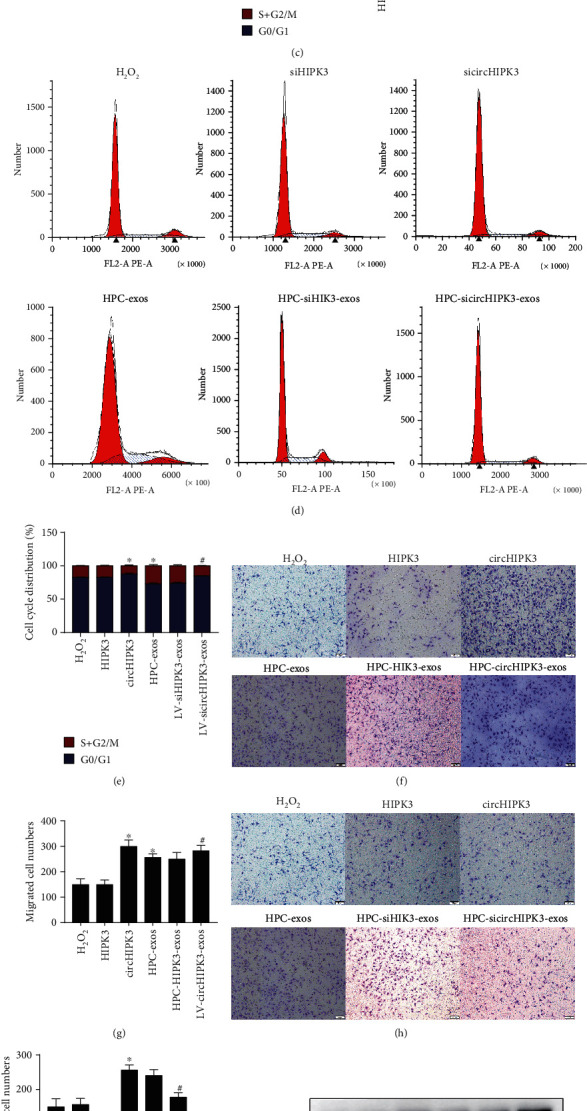
HPC-exosomal circHIPK3 accelerates cell cycle progression and migration of cardiac endothelial cells. circHIPK3, circHIPK3 siRNA, linear HIPK3, or linear HIPK3 siRNA was transfected into cardiac endothelial cells for 48 h to evaluate cell proliferation and migration. Furthermore, cardiac endothelial cells were incubated with HPC-HIPK3-exos, HPC-circHIPK3-exos, HPC-siHIPK3-exos, or HPC-sicircHIPK3-exos to understand the role of exosomal circHIPK3. (a) circHIPK3 expression in cardiac endothelial cells subjected to different treatments was analyzed with qPCR. (b, d) Flow cytometry was performed to analyze the distribution of cells in the cell cycle. (c, e) Representative quantification of the data from (b) and (d), respectively. (f, h) Cell migration among the six groups as confirmed by the Transwell assay (lower, bars = 50 *μ*m). (g, i) The number of migrated cells per field was determined. (j, l) Western blot analyses were performed. (k, m) Quantification of the relative protein levels of cyclinD1 and PCNA in cardiac endothelial cells was performed (means ± SD, *n* = 3, ^∗^*P* < 0.05, compared with the H_2_O_2_ group, ^#^*P* < 0.05 compared with the HPC-exo group).

The above data indicated that exosomes derived from CMs subjected to hypoxia have a greater capacity to promote cardiac endothelial cell proliferation than exosomes derived from CMs under normoxic conditions. Exosomes facilitate the communication between adjacent cells by transferring circRNAs [26], and circRNA expression is dynamically regulated after cells are exposed to hypoxia [27]. In a previous study, we demonstrated that circHIPK3 was enriched in exosomes derived from CMs cultured under hypoxic conditions [11]. This prompted us to further investigate the function of exosomal circHIPK3 on the proliferation and migration of cardiac endothelial cells and neovascularization. To understand the role of exosomal circHIPK3 in cardiovascular biology, we first detected circHIPK3 expression in cardiac endothelial cells incubated with HPC-exos or Nor-exos by using qPCR. The results indicate that compared with the control group, the H_2_O_2_ group exhibited significantly downregulated levels of circHIPK3. Compared with H_2_O_2_ treatment alone, exosome pretreatment significantly rescued circHIPK3 levels, and circHIPK3 expression was higher in the HPC-exo group than the Nor-exo group (Figure 3(a)).

To investigate the role of circHIPK3 alone in cardiac endothelial cells, we generated lentiviral vectors that expressed ectopic circHIPK3 or siRNA targeting circHIPK3 (sicircHIPK3) and transfected these vectors into mouse cardiac endothelial cells for 48 h. The impact of linear HIPK3 was also determined by conducting gain- and loss-of-function analyses. Cell proliferation resulting from altered cell cycle progression is a main event in neovascularization. In cardiac endothelial cells under oxidative conditions, circHIPK3 overexpression significantly promoted the cell cycle, as evidenced by an accelerated G1/S transition in cells assessed by flow cytometry. Suppressing circHIPK3 arrested the cell cycle at G0/G1 phase (Figures 3(b)–3(e)). Furthermore, the migration ability of cells was significantly increased in the circHIPK3 overexpression group and further decreased in the circHIPK3 suppression group compared with the H_2_O_2_ group (Figures 3(f)–3(i)). In addition, the levels of cyclinD1 and PCNA were upregulated in cells overexpressing circHIPK3 but downregulated in cells with circHIPK3 suppression (Figures 3(j)–3(m)). However, neither overexpressing nor inhibiting linear HIPK3 expression had a significant effect on cell proliferation and migration (Figures 3(a)–3(m)).

To further verify whether the effects of HPC-exos on cardiac endothelial cells were dependent on circHIPK3 but not linear HIPK3, we overexpressed and inhibited both circHIPK3 and linear HIPK3 in hypoxia-induced CMs by transfecting them with HIPK3 siRNA, linear HIPK3, circHIPK3, or circHIPK3 siRNA for 48 h and then culturing them under hypoxic conditions for 12 h. Their exosomes (HPCHIPK3-exos, HPC-circHIPK3-exos, HPC-siHIPK3-exos, and HPC-sicircHIPK3-exos, respectively) were cocultured with cardiac endothelial cells. There is no doubt that HPC-exos significantly induced increases in cyclinD1 and PCNA and promoted cell cycle progression and cell migration (Figures 3(j)–3(m)). HPC-exos derived from CMs with circHIPK3 overexpression induced enhanced cell cycle progression and migration of cardiac endothelial cells (Figures 3(b)–3(i)). However, HPC-exos derived from CMs with inhibited circHIPK3 expression resulted in reduced levels of cell cycle progression and migration in cardiac endothelial cells (Figures 3(b)–3(i)). The overexpression or inhibition of linear HIPK3 did not change the effects of HPC-exos on the cell cycle or migration (Figures 3(b)–3(i)). The use of HPC-exos is a potential strategy for promoting cell cycle progression and migration of cardiac endothelial cells under oxidative stress conditions by rescuing downregulated circHIPK3 expression.

### 3.4. miR-29a Is Upregulated in Cardiac Endothelial Cells and Suppresses Cell Proliferation and Migration by Targeting VEGF In Vitro

Previous studies have shown that circHIPK3 can regulate cell growth by acting as a miR-29a inhibitor [28]. In our earlier work, we verified that miR-29 and circHIPK3 colocalize in the cytoplasm in cardiac endothelial cells and can directly bind to each other to regulate cell function. miR-29a was shown to be upregulated in endothelial cells under oxidative stress, which subsequently promoted apoptosis. Thus, the effect of miR-29a could be effectively inhibited by overexpressing circHIPK3 in cells.

**Figure 4:**
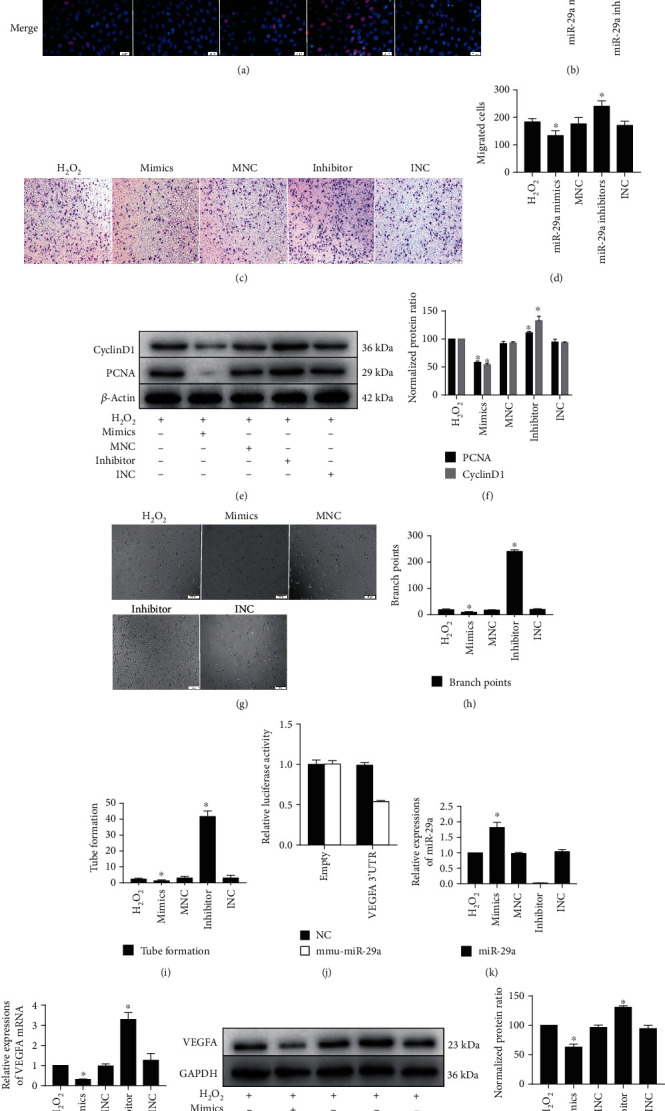
miR-29a regulated the proliferation, migration, and tube formation of cardiac endothelial cells by targeting VEGFA. miR-29a mimics or inhibitors were transfected into cardiac endothelial cells via Lipofectamine 2000; 48 h later, an oxidative stress model was established with the cardiac endothelial cells to investigate the effects of miR-29a. (a) Cell proliferation of cardiac endothelial cells as determined by EdU assays. (b) Quantitative analysis of (a). (c) Migration of cardiac endothelial cells was measured by Transwell migration assays (scale bar = 50 *μ*m). (d) The number of migrated cells per field was determined. (e) Western blot analyses were performed. (f) Relative protein levels of cyclinD1 and PCNA in cardiac endothelial cells were determined. (g) Representative images of cardiac endothelial cells in Matrigel. (h, i) Quantitative assessment of the total number of meshes and branch points. (j) The dual-luciferase activity was verified by cotransfecting either miR-29a mimics or miR-29a-NC and the luciferase reporter vectors pmiRGLO-VEGFA-Mut or pmiRGLO-VEGFA-WT. (k) Relative miR-29a expression in different groups of cardiac endothelial cells was detected via qPCR analysis. (l) VEGFA mRNA expression in cardiac endothelial cells in different groups was also detected by using qPCR analysis. (m) Western immunoblotting was used to detect the relative protein expression of VEGFA. (n) Quantitative analysis of relative VEGFA protein expression (mean ± SD, *n* = 3; ^∗^*P* < 0.05, compared with the H_2_O_2_ group).

To investigate the role of miR-29a in cardiac endothelial cells, we treated cells with miR-29a mimic or inhibitor and evaluated their effects on angiogenesis. The data showed that compared with the H_2_O_2_ group, the miR-29a mimic group showed decreased proliferation and migration, as determined by the EdU and Transwell assays, respectively (Figures 4(a)–4(d)). By contrast, inhibiting miR-29a significantly promoted proliferation and migration of cardiac endothelial cells, even under oxidative stress conditions (Figures 4(a)–4(d)). The relative quantities of cyclinD1 and PCNA were also analyzed by Western blotting. CyclinD1 and PCNA were substantially decreased in the miR-29a mimic group compared with the H_2_O_2_ group, while they were substantially increased in the miR-29a inhibitor group (Figures 4(e) and 4(f)). In addition, we also evaluated tube formation. The results showed that the relative tube formation and numbers of branch points were decreased in the miR-29a mimic group compared with the H_2_O_2_ group, while they were increased in the miR-29a inhibitor group compared with the H_2_O_2_ group (Figures 4(g) and 4(h)).

Subsequently, upon searching the miRanda database, we found that the 3′-UTR of VEGFA contains a binding site of miR-29a. Angiogenesis is mediated via activation of VEGFR2 by its primary ligand, VEGFA; VEGFR2 is then phosphorylated (p-VEGFR2) and translocates to the nucleus [29]. In addition, miR-29a has been shown to play a key antiangiogenic role within the tumor microenvironment by suppressing the expression of VEGFA [30]. Dual-luciferase reporter assays were used to confirm that VEGFA is a target gene of miR-29a in cardiac endothelial cells (Figure 4(j)). In addition, the miR-29a mimics effectively increased miR-29a expression in cardiac endothelial cells whereas the miR-29a inhibitor effectively reduced it (Figure 4(k)). We also found that VEGFA mRNA levels were significantly reduced in the miR-29a mimic group, whereas the miR-29a inhibitor group showed the opposite trend (Figure 4(l)). Western blot analysis revealed that the miR-29a inhibitor effectively promoted VEGFA expression, while the miR-29a mimics clearly reduced VEGFA levels (Figures 4(m) and 4(n)). These results suggested that miR-29a targets VEGFA and inhibits its expression. Therefore, the effects of miR-29a on inhibiting the proliferation, migration, and tube formation of cardiac endothelial cells were partially mediated by targeting VEGFA.

### 3.5. HPC-Exosomal CircHIPK3 Induces Cell Proliferation, Migration, and Tube Formation via miR-29a/VEGFA in Cardiac Endothelial Cells

**Figure 5:**
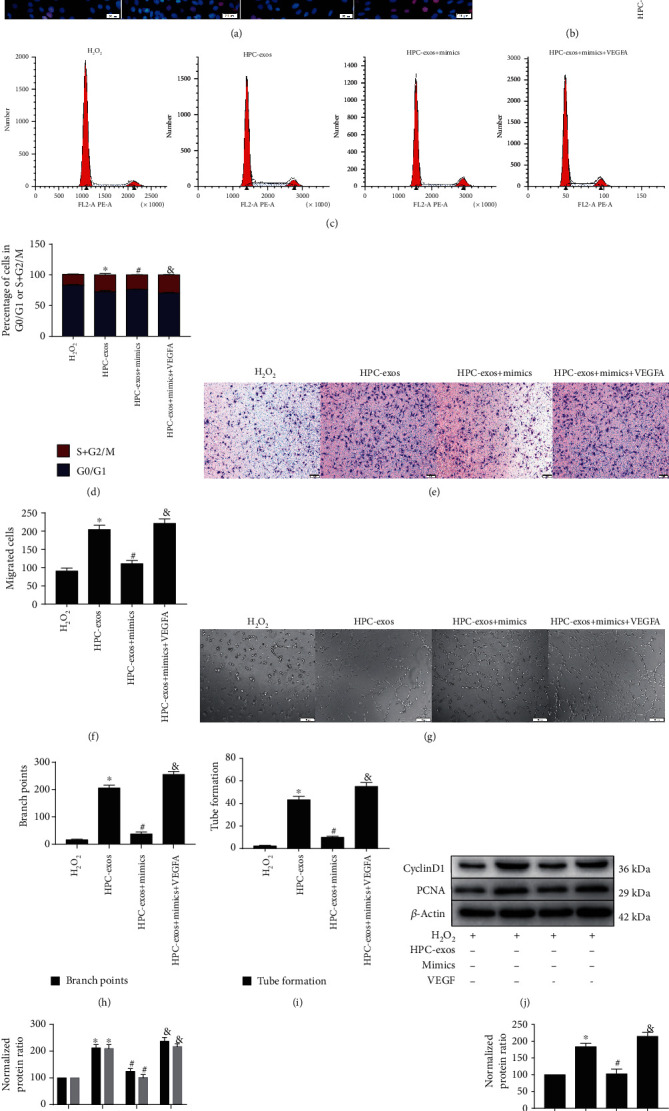
Exosomal circHIPK3 induces cell proliferation, migration, and tube formation via the miR-29a/VEGFA axis in cardiac endothelial cells. To further investigate the role of HPC-exosomal circHIPK3, miR-29a or VEGFA was transfected into cardiac endothelial cells. (a) Proliferation of cardiac endothelial cells was determined by EdU assays. (b) Quantitative analysis of (a). (c) Flow cytometry was performed to analyze the cell cycle distribution. (d) Representative histograms are shown. (e) Migration of cardiac endothelial cells was measured by Transwell migration assays (scale bar = 50 *μ*m). (f) The number of migrated cells per field was determined. (g) Representative images of cardiac endothelial cells in Matrigel. (h, i) The total number of meshes and branch points were evaluated with a quantitative analysis method. (j) Western blot analyses were performed. (k) Relative protein levels of cyclinD1 and PCNA in cardiac endothelial cells were determined. (l) VEGFA protein expression was detected by immunoblotting. (m) VEGFA protein relative expression was evaluated with quantitative analysis method (mean ± SD, *n* = 3, ^∗^*P* < 0.05, compared with the H_2_O_2_ group).

To investigate whether HPC-exos mediated angiogenesis in cardiac endothelial cells by targeting VEGFA via inhibition of miR-29a, cardiac endothelial cells were transfected with miR-29a mimics for 48 h and then exposed to HPC-exos for 24 h. EdU and Transwell assays revealed that the miR-29a mimics reversed the effects of HPC-exos on the levels of proliferation and migration in cardiac endothelial cells (Figures 5(a)–5(d)). In addition, flow cytometry showed that the facilitated G1/S transition induced by HPC-exos was also reversed by miR-29a mimics (Figures 5(e) and 5(f)). Moreover, transfection of miR-29a mimic in cardiac endothelial cells could also partly block the HPC-exo-induced increase in tube formation capacity and the expression of the proliferation-related proteins cyclin D1 and PCNA (Figures 5(g) and 5(h)). Western blot analysis also showed that in the HPC-exo+mimic+VEGFA group, miR-29a significantly inhibited VEGFA expression, while VEGFA overexpression strikingly reversed the reduced VEGFA levels (Figures 5(l) and 5(m)). In addition, we observed that exogenous VEGFA could partially reverse the repressive effect of miR-29a on the cell proliferation, migration, and tube formation of cardiac endothelial cells (Figures 5(a)–5(k)). In conclusion, in cardiac endothelial cells, HPC-exos promoted angiogenesis by regulating the cell cycle, cell proliferation, and migration through circHIPK3/miR-29a/VEGFA signaling axes in vitro and in vivo.

## 4. Discussion

It is well established that there is tightly regulated crosstalk among the different cell types via CM-derived exosomes. For example, exosomes secreted by CMs subjected to ischemia promote cardiac angiogenesis by delivering miRNA-222 and miRNA-143 [10]. The regulatory effect of CM-derived exosomes in endothelial cells is relatively well studied. However, the role of a few exosomal circRNAs in this interplay remains unknown. CircRNAs can be enriched in exosomes under certain pathological conditions; their capture by neighboring cells regulate the function of these target cells [31]. In our previous study, we found that exosomal circHIPK3 released from hypoxia-induced CMs could inhibit miR-29a activity and then regulate oxidative damage in cardiac endothelial cells in the microvasculature, thus leading to increased IGF-1 expression in vitro [11]. In the current study, we revealed that the exogenous delivery of exosomes secreted by CMs subjected to hypoxia can play an important role in promoting angiogenesis after MI in vitro and in vivo. At the same time, we also confirmed that circHIPK3 was the most enriched circRNA in HPC-exos, inducing the angiogenic process.

Hypoxia could increase myocardial tolerance as an adaptive response to prevent endoplasmic reticulum stress and apoptosis [32]. However, hypoxic preconditioning enhanced the benefits of cell-derived exosomes in an animal MI model and led to an increase in the proangiogenic effect of exosomes [33]. In this study, we also observed that exosomes released from hypoxic CMs could promote angiogenesis after an acute MI and improve myocardial fibrosis. It was verified that HPC-exos promote migration, proliferation, and tube formation of cardiac endothelial cell in vitro; however, the exact mechanism is unclear.

Exosomes are vectors of circRNAs, lncRNAs, and miRNAs; they serve as a mechanism that allows cell-to-cell communication [20, 31]. CircRNAs can compete for miRNA- or protein-binding sites to regulate several diseases [34]. Interestingly, some circRNAs are differentially expressed in cells subjected to hypoxic conditions [27].

CircHIPK3 is expressed in endothelial cells and can increase cell proliferation and improve vascular dysfunction [16]. Our previous study found that circHIPK3 within HPC-exos could regulate the oxidative stress damage of cardiac endothelial cells in the microvasculature through the miR-29a/IGF-1 axis [11]. However, another study showed that circHIPK3 overexpression had the opposite effect [13]. On the basis of our previous results and those of others, we hypothesized that circHIPK3 released from HPC-exos affects cardiac endothelial cells, resulting in angiogenesis. In the present study, we observed upregulated levels of circHIPK3 in cardiac endothelial cells treated with exosomes from MCs cultured under oxidative conditions. We also showed that overexpression of circHIPK3 alone, but not of linear HIPK3, promoted the proliferation, migration, and tube formation of cardiac endothelial cells. Knocking down circHIPK3 but not linear HIPK3 mRNA suppressed the proliferation, migration, and tube formation of cardiac endothelial cells. Moreover, when circHIPK3 was overexpressed in CMs subjected to hypoxic conditions, the results suggested that HPC-exos from these CMs enhanced the cell cycle progression and migration of cells. However, HPC-exos derived from CMs with inhibition of circHIPK3 reduced the cell cycle progression and migration. The overexpression or inhibition of linear HIPK3 mRNA in HPC-exos did not change the effect of HPC-exos on either cell cycle or migration. Taken together, the results suggest that circHIPK3 from HPC-exos derived from CMs mediates cardiac endothelial cell angiogenesis.

In corroboration with a recent study suggesting that some circRNAs have miRNA binding sites and thus can sequester homologous miRNAs using these sites [35], our previous studies stated that circHIPK3 and miR-29a are colocalized in the cytoplasm of cardiac microvascular endothelial cells and that circHIPK3 interacts with miR-29a and decreases miR-29a stability [11]. Based on this, we also assessed the effect of miR-29a on cardiac endothelial cell angiogenesis by establishing the miR-29a gain-of-function and loss-of-function models. Our data showed that the miR-29a inhibitor effectively accelerated cell cycle progression and increased the migration of cardiac endothelial cells and followed by enhanced tube formation. Moreover, these effects were strikingly similar to the effects of circHIPK3 overexpression.

Canonically, miRNAs can decrease the expression of a target protein by either inhibiting translation or inducing the degradation of its mRNA [36]. Many target mRNAs of miR-29a, including but not limited to VEGFA in gastric carcinoma [30] and IGF-1 in cardiac endothelial cells [11], have been discovered. We investigated VEGFA, which has been shown to play a primary role in various cellular processes, including cell cycle progression and angiogenesis [37]. Moreover, cardiac-specific deletion of VEGFA in mice resulted in hypovascular hearts with basal contractile dysfunction [38]. We also found that upregulated miR-29a levels effectively decreased the expression of VEGFA mRNA and protein by binding to the 3′-UTR of VEGFA mRNA. miR-29a mimics abrogated the HPC-exo-mediated protective effects by decreasing cell proliferation and migration under oxidative conditions. By contrast, overexpression of VEGFA significantly increased proliferation and migration in cardiac endothelial cells transfected with miR-29a mimics. The modulation of miR-29a levels in our study revealed a crucial role of HPC-exosomal circHIPK3/miR-29a in the regulation of VEGFA levels, suggesting that exosomal circHIPK3 is a molecular regulator of cardiac function via miR-29a/VEGFA signaling.

**Figure 6:**
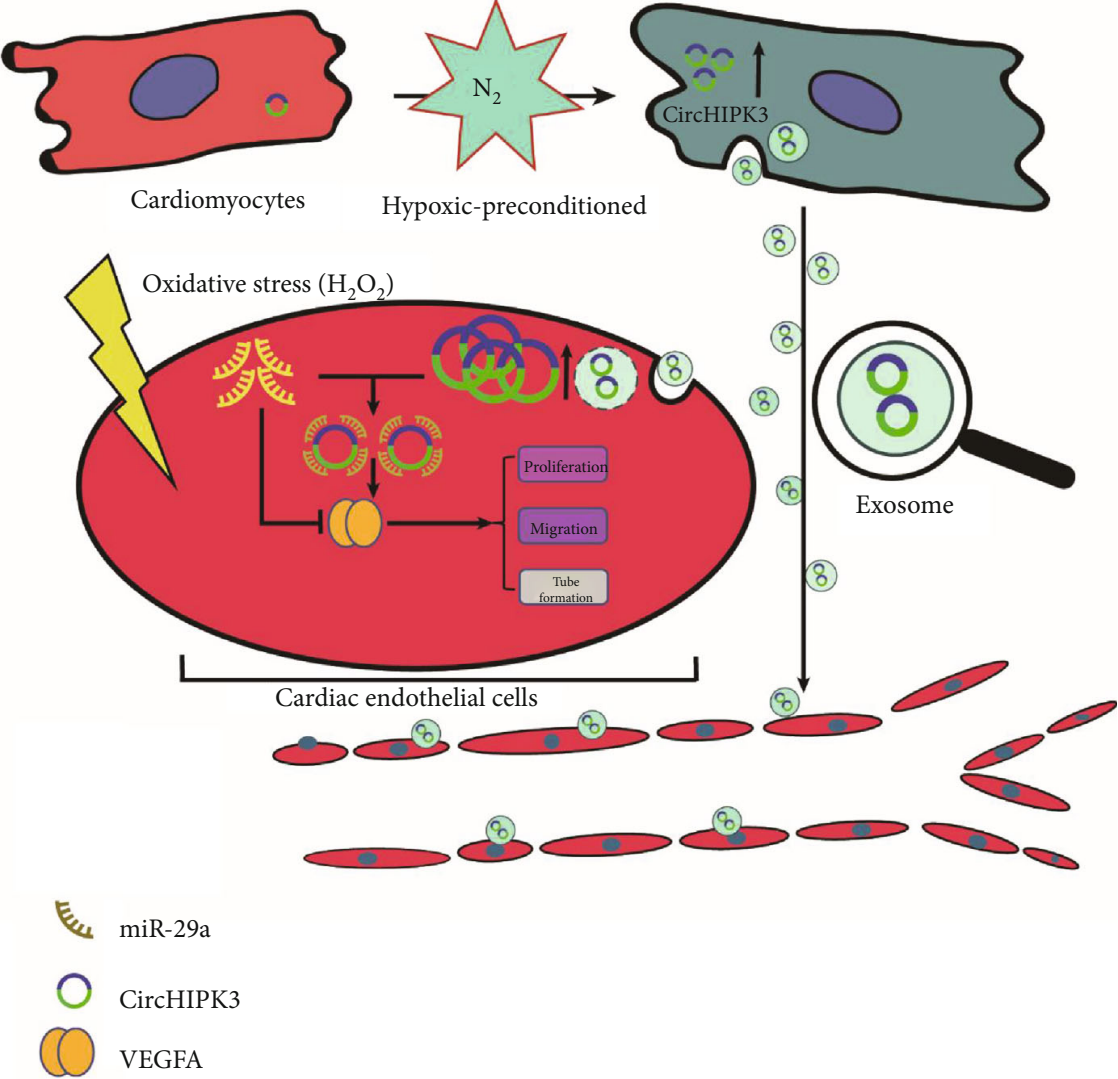
A schematic cartoon of the circHIPK3/miR-29g/VEGFA axis in cardiac endothelial cells. CircHIPK3 is upregulated in CMs subjected to hypoxia and released to the extracellular space by exosomes. Subsequently, adjacent cardiac endothelial cells internalize these exosomes, which causes circHIPK3 levels to increase in cardiac endothelial cells. CircHIPK3 could inhibit miR-29a activity and then regulate angiogenesis in cardiac endothelial cells by increasing VEGFA expression.

In summary, our data demonstrate that HPC-exos derived from CMs exhibit cardioprotective effects by enhancing neovascularization, limiting the infarct size, and preserving post-MI cardiac endothelial cell function and integrity in part through the miR-29a/VEGFA signaling axis (Figure 6). To the best of our knowledge, we show that the use of HPC-exos derived from CMs is a feasible approach to limit ischemic injury.

## Figures and Tables

**Table 1:** The sequences of primers used for PCR.

Primer sequence	
CircHIPK3	Forward 5′- GGATCGGCCAGTCATGTATC-3′ Reverse 5′-ACCGCTTGGCTCTACTTTGA-3′
HIPK3 mRNA	Forward 5′- GTGATCCGGCCTGTTCTTCA-3′ Reverse 5′- TGACTGGCCGATCCAAAGTC-3′
GAPDH mRNA	Forward 5′-GTCAAGGCTGAGAACGGGAA-3′ Reverse 5′-AAATGAGCCCCAGCCTTCTC-3′
*β*-Actin mRNA	Forward 5′-TTGTTACAGGAAGTCCCTTGCC-3′ Reverse 5′-ATGCTATCACCTCCCCTGTGTG-3′
VEGFA mRNA	F: 5′-ATGATTCTGCCCTCCTCCTT-3′ R: 5′-CCTTGCTGCTCTACC TCCAC-3′
miR-29a	RT: 5′-GTCGTATCCAGTGCGTGTCGTGGAGTCGGCAATTGCACTGGA TACGACTAACCGAT-3′ F: 5′-ACACTCCAGCTGGGTAGCACCATCTGAAAT-3′ R: 5′-TGGTGTCGTGGAGTCG-3′
U6	RT: 5′-AACGCTTCACGAATTTGCGT-3′ F: 5′-CTCGCTTCGGCAGCACA-3′ R: 5′-AACGCTTCACGAATTTGCGT-3′

## Data Availability

The data used to support the findings of this study are included within the article.
